# Selection and Characterization of a DNA Aptamer That Can Discriminate between cJun/cJun and cJun/cFos

**DOI:** 10.1371/journal.pone.0101015

**Published:** 2014-06-26

**Authors:** Ryan D. Walters, David T. McSwiggen, James A. Goodrich, Jennifer F. Kugel

**Affiliations:** Department of Chemistry and Biochemistry, University of Colorado, Boulder, Colorado, United States of America; University of Essex, United Kingdom

## Abstract

The AP-1 family of transcriptional activators plays pivotal roles in regulating a wide range of biological processes from the immune response to tumorigenesis. Determining the roles of specific AP-1 dimers in cells, however, has remained challenging because common molecular biology techniques are unable to distinguish between the role of, for example, cJun/cJun homodimers versus cJun/cFos heterodimers. Here we used SELEX (systematic evolution of ligands by exponential enrichment) to identify and characterize DNA aptamers that are >100-fold more specific for binding cJun/cJun compared to cJun/cFos, setting the foundation to investigate the biological functions of different AP-1 dimer compositions.

## Introduction

The activator protein-1 (AP-1) family of transcriptional activators consists of dimeric combinations of Jun and Fos proteins that regulate a variety of transcriptional programs in response to various stimuli [1]. AP-1 proteins share a common basic leucine zipper (bZip) motif that is responsible for dimerization and DNA-binding. AP-1 proteins recognize the AP-1 DNA binding site (consensus sequence: 5′-TGA(C/G)TCG-3′), also known as a phorbol 12-O-tetradecanoate-13-acetate (TPA) response element (TRE). Different subsets of AP-1 proteins have differing dimerization requirements. cJun, for example, can homo- and heterodimerize while cFos can only form heterodimers. These AP-1 dimers regulate a wide variety of cellular processes including the immune response, cell proliferation, apoptosis, and tumorigenesis [2].

The role of AP-1 proteins has been widely studied; however, discerning the distinct roles of individual dimer compositions remains challenging. Functions unique to cJun homodimers, but not cJun/cFos heterodimers have been identified. For example, cJun homodimers are not only capable of binding cis-elements on DNA to activate transcription but can also function as transcriptional co-activators by binding directly to other DNA-bound transcription factors, such as NFATc2 and PU.1 [3]–[5]. This function is unique to cJun/cJun and does not occur with cJun/cFos. Additionally, by expression of dimer specific mutants it was shown that cJun/cJun, cJun/Fra2, and cJun/ATF2 dimers have distinct functions in cJun induced transformation of chicken embryo fibroblasts [6]. Specifically, cJun/Fra2 induces anchorage independence and cJun/ATF2 induces growth factor independence. Another strategy to delineate unique functions of AP-1 dimers employed covalently tethering different combinations of Jun and Fos partners and testing their activities in cells. Different dimer compositions showed promoter-specific differences in activating transcription of reporter genes [7], [8]. Together, these observations underscore the importance of developing tools to distinguish between different AP-1 dimer compositions in cells.

Current strategies of gene knockout, siRNA knockdown, and transcription factor decoys have provided substantial insight into the role of AP-1 proteins in response to various stimuli [2], [9]–[11]. These strategies, however, do not discern the biological functions of different dimer compositions containing the same protein. For example, an AP-1 DNA decoy, which is an exogenous oligonucleotide containing the consensus AP-1 site, can sequester AP-1 proteins from gene promoters; however, this decoy targets all AP-1 dimers regardless of their composition. Moreover, knocking down cJun will inhibit the function of cJun/cJun homodimers as well as cJun heterodimers such as cJun/cFos. Similarly, ChIP assays against cJun cannot distinguish between sites of homo and heterodimer occupancy. Given the importance of AP-1 dimer composition on biological processes, research tools that allow us to discern between AP-1 dimers with different compositions would be very useful.

SELEX (systematic evolution of ligands by exponential enrichment) is an iterative selection process to identify aptamers from a large DNA or RNA library that bind the desired target [12], [13]. Here, we used SELEX to isolate a DNA aptamer that binds cJun; biochemical experiments found that the aptamer has high affinity and specificity for cJun/cJun homodimers compared to cJun/cFos heterodimers. The secondary structure and minimal binding region of the aptamer was determined. Using this aptamer we are able to specifically block cJun/cJun homodimers from binding AP-1 DNA. Moreover, the aptamer is capable of blocking cJun/cJun from cooperatively binding DNA with NFATc2, a common transcriptional partner of AP-1 proteins. We demonstrate that in cells this aptamer represses cJun/cJun-dependent IL-2 reporter activity. This work provides the foundation and selection method for specifically targeting different AP-1 dimers, which has the potential to provide insight into the unique biological roles of distinct AP-1 complexes.

## Materials and Methods

### Plasmid construction

The pET-cJun, pET-\is-cFos, pET-NFAT(DBD), and IL2-firefly-Luc reporter plasmids have been previously described [5], [14]. To prevent aggregation in EMSA, a cysteine at position 269 in cJun and 154 in cFos were mutated to serine by QuickChange site directed mutagenesis.

### Protein purification

cJun was expressed and purified as previously described [14]. The NFAT DNA-binding-domain was expressed and purified as previously described [5]. For cFos expression and purification, cultures co-transformed with pET-6His-cFos and pSBET were grown in the presence of 100 µg/ml ampicillin and 50 µg/ml kanamycin in Luria-Bertani broth at 37°C to an optical density of 0.4 at 600 nm before expression was induced by the addition of isopropylthio-β-D-galactoside at a final concentration of 0.5 mM. After 2 hr, cells were harvested and the cell pellet was resuspended in a buffer containing 20 mM Tris (pH 7.9), 1 mM EDTA, 100 mM NaCl, 1 mM DTT, 0.2 mM phenylmethylsulfonyl fluoride (PMSF), and sonicated 4 times for 15 s. Samples were centrifuged for 30 min at 15,000 rpm and 4°C. Precipitated material containing 6His-cFos was resuspended in 10 ml of 20 mM Tris (pH 7.9), 1 mM EDTA, 100 mM NaCl, 5 mM DTT, 0.2 mM PMSF and sonicated 2 times for 30 s. The pellet was washed three more times by resuspending in 10 ml of 20 mM Tris (pH 7.9), 1 mM EDTA, 100 mM NaCl, 5 mM DTT, 0.2 mM PMSF followed by centrifugation. The pellet from the final wash was resuspended in 10 ml of buffer A (20 mM Tris (pH 7.9), 1 mM EDTA, 5 mM DTT, and 8 M urea, 0.1 M NaCl) containing 20 mM imidazole. Soluble material was loaded onto a Ni-NTA agarose column (Qiagen) and washed with 5 column volumes of buffer A containing 20 mM imidazole followed by 5 column volumes of buffer A containing 40 mM imidazole. cFos was eluted with buffer A containing 500 mM imidazole. Purified cFos was mixed with equimolar purified cJun in buffer A and subjected to three sequential dialyses in buffer B (20 mM Tris (pH 7.9), 0.1 mM EDTA, 10% glycerol, 5 mM DTT) containing the following additions: 1) 1 M urea and 1 M NaCl; 2) 1 M NaCl; 3) 0.1 M NaCl. After dialysis, the sample containing purified cJun/cFos heterodimers was separated into aliquots and stored at −80°C.

### In vitro selection

The ssDNA SELEX pool (IDT) was designed with a 40 nt randomized region flanked by the 5′ constant region 5′-GGGAGATCACTTACGGCACC-3′ and the 3′ constant region 5′-CCAAGGCTCGGGACAGCG-3′. Immobilized AP-1 DNA was made using biotin-5′-AGGTCGTGACTCAGCGG-3′ annealed to 5′-CCGCTGAGTCACGACCT-3 (the AP-1 site is underlined). 1 µmol double stranded DNA was incubated with 25 µl magnetic streptavidin beads (Invitrogen). Beads were washed three times with binding buffer (20 mM Tris (pH 7.9), 0.1 mM EDTA, 10% glycerol, 1 mM DTT, 150 mM KCl, 5 mM MgCl_2_, 0.04% NP-40). cJun (94 pmol) and 1µg poly(dI•dC) were added to the immobilized AP-1 DNA and incubated at room temperature with nutation for 25 min in binding buffer. The poly(dI•dC) served as a non-specific competitor to minimize background partitioning of the SELEX pool and any non-specific interactions that might occur between cJun and the DNA or beads. Unbound cJun was removed by washing the beads three times with binding buffer.

For the initial round of SELEX, 1.67 nmol of a ssDNA pool consisting of ∼1×10^15^ sequences was added in a volume of 3.4 µl to 150 µl of DNA-bound cJun beads and incubated for 30 min at room temperature in binding buffer with nutation. Unbound aptamers were removed by three 1 min washes in 100 µl binding buffer with nutation. ssDNA sequences were eluted by addition of 150 µl elution buffer (20 mM Tris (pH 7.9), 1.5 M NaCl) for 10 min at room temperature with nutation, then desalted using a G-25 column (GE Lifesciences). The ssDNA sequences in the eluates were amplified by PCR (using 25 µl of eluate) with forward primer 5′-FAM-CGGGAGATCACTTACGGCACC-3′ and reverse primer 5′-AAAAAAAAAAAAAAAAAA-iSp9-CGCTGTCCCGAGCTTTGG-3′ complementary to the constant regions. The poly-A stretch in the reverse primer added a poly-A tail to the strand complementary to the aptamer pool; iSp9 is a triethylene glycol spacer (IDT). The ssDNA aptamer pool was separated and purified from its complementary strand containing the poly-A stretch using a 8% denaturing polyacrylamide gel. Four rounds of positive selection were conducted using the conditions described for the first round with extension of the washing incubation to 2 min each for round 3, and 3 min each for round 4. This was followed by 2 rounds which included a negative selection against immobilized AP-1 DNA and washing incubation times of 3 and 5 min for rounds 5 and 6, respectively. For the negative selection the ssDNA SELEX pool was incubated with beads containing immobilized AP-1 DNA (30 µl beads for round 5 and 60 µl of beads for round 6) for 30 min in binding buffer at room temperature with nutation. The unbound ssDNA pool was then transferred to a tube containing 25 µl beads with DNA-bound cJun and the positive selection was performed as described. Aptamers from the final round of SELEX were amplified using a forward primer 5′-GGGAGCTCACTTACGGCACC-3′ containing a SacI restriction site and a reverse primer 5′-GCCAAGCTTCGCTGTCCCGAGCCTTGG-3′ containing a HindIII restriction site. The PCR product was digested with SacI and HindIII, gel purified, and ligated into a pUC18 vector for sequencing.

### Electrophoretic mobility shift assays

5′-FAM or ^32^P-labeled aptamer or AP-1 DNA and purified cJun/cJun, cJun/cFos, and/or NFAT DBD were incubated together in 20 µl buffer containing 100 mM KCl, 10% glycerol, 4 mM MgCl_2_, 20 mM Tris (pH 7.9), 20 mM HEPES (pH 7.9), 0.1 mM DTT, 60 µg/ml BSA, and 0.06% NP-40 on ice for 20 min. For AP-1 DNA, the following oligos were annealed: 5′-AGGTCGTGACTCAGCGG-3′ and 5′-CCGCTGAGTCACGACCT-3 (the AP-1 site is underlined). 50 ng Poly(dI•dC) was added to each reaction and incubated for an additional 5 min on ice. The reactions were subjected to electrophoresis through 4% polyacrylamide gels containing 1X Tris-glycine and 5% glycerol. For K_D_ determination the ^32^P-labeled DNA concentration was held constant at 50 pM and the protein was titrated over the indicated concentrations; data were fit with the following equation: Fraction bound = Fraction bound_max_([cJun/cJun]/(K_D_+[cJun/cJun])). For the EMSA with the round 6 pool the DNA concentration was held constant at 1 nM. The bands were visualized by fluorescent scanning (Typhoon 9400) for FAM-labeled DNA or phosphorimaging (Typhoon 9400) for ^32^P-labeled DNA. Data were quantified using ImageJ software. The sequences of the AP-1 DNA decoy and mutant AP-1 decoy are as follows: AP-1 DNA 5′-GTCCATGACTCAGAAGAGACACACTCTTCTGAGTCATGGAC-3′ (AP-1 sequence underlined) mutant AP-1 decoy 5′-GTCCAAATCTCAGAAGAGACACACTCTTCTGAGATTTGGAC-3′ (mutant AP-1 sequence underlined).

### Nuclease probing and hydroxyl radical footprinting

20 fmol of ^32^P-labeled aptamer-19 was folded in RM buffer (20 mM HEPES (pH 7.9), 100 mM KCl, 8 mM MgCl_2_) by heating to 95°C and cooling on ice. Refolded aptamer was incubated with 30 units S1 nuclease in 1X S1 nuclease buffer (Promega) in a 20 µl reaction for 1 min on ice before adding 80 µl stop mix (200 mM KCl, 50 mM EDTA, 0.3 µg/µl yRNA). DNA was phenol-chloroform extracted and ethanol precipitated prior to resolving on a 10% denaturing polyacrylamide gel. Bands were visualized by phosphorimaging and quantified using ImageJ software. For the CviKI-1 digest, 20 fmol of ^32^P-labeled aptamer-19 folded in RM buffer was digested in 1X NEB4 buffer with 20 units CviKI-1 for 1 hr at room temperature. DNA was phenol-chloroform extracted and ethanol precipitated prior to resolving on a 10% denaturing polyacrylamide gel. Bands were visualized by phosphorimaging. The secondary structure drawing of aptamer-19 was created using VARNA software [15].

For hydroxyl radical footprinting, glycerol was removed from the cJun prep using a G-25 column (GE lifescience). Protein was incubated with 20 fmol of folded ^32^P-labeled aptamer-19 in buffer containing 20 mM Tris (pH 7.9), 100 mM KCl, 4 mM MgCl_2_, 0.1 mM DTT, 20 µg/µl BSA, 0.06% NP-40, and 2.5 ng/µl Poly(dI•dC) for 20 min on ice. Cleavage was initiated by mixing 1 µl each of 10 mM Fe(II)EDTA, 0.6% H_2_O_2_, and 10 mM sodium ascorbate for 3 min followed by quenching with 1 µl of 100 mM thiourea. Samples were ethanol precipitated and resolved on a 11.3% denaturing polyacrylamide gel. Bands were visualized by phosphorimaging and quantified using the software ImageJ.

### Transfection assays

COS-7 cells (purchased from ATCC) were maintained at 37°C and 5% CO_2_ in DMEM containing 10% FBS, 100 U/ml penicillin, 100 µg/ml streptomycin, and 2 mM GlutaMax. On the day of the transfection the cells were 75% confluent. Cells were transfected by Neon electroporation according to the manufacturers instructions (Invitrogen). Briefly, 500 ng of each protein expression construct, IL2-firefly-Luc reporter [14], and pRL-TK-Renilla-luciferase (Promega) were mixed with the either aptamer-19 or its antisense sequence (AS) as a control; the oligos contained phosphorothioate linkages at the three terminal positions on both ends to increase cellular stability. Scrambled DNA (oligo of the same chemical composition as aptamer-19, but with a different sequence; 5′-GTGACACGAATTGGGACCAGCGTATGGCTGATATAACATGTTTCGACCGAGCCTGACCGGTTG-3′) was used to maintain an equal amount of oligonucleotide DNA for each reaction. Cells were electroporated and seeded into individual wells of a 6-well plate in the absence of antibiotics. 16 hr post transfection, cells were stimulated with 1 µM ionomycin and 20 ng/ml PMA for 6 hr. Cells were then harvested and lysed with 250 µl Passive Lysis Buffer (Promega). Firefly and renilla luciferase activities were determined using the Dual-luciferase kit (Promega).

## Results

### SELEX targeting DNA-bound cJun homodimers yields aptamers that bind cJun/cJun with high affinity and specificity

**Figure 1:**
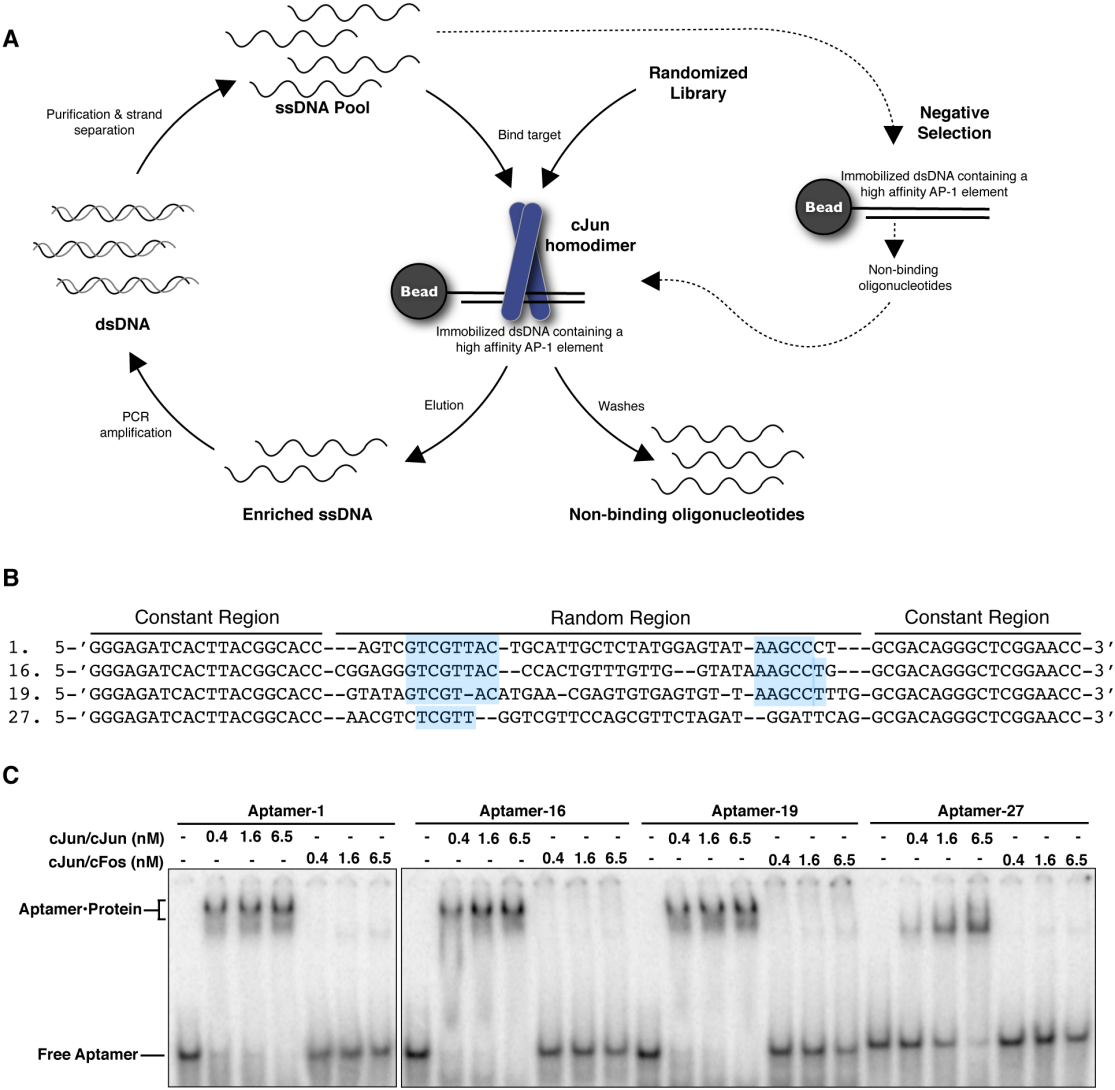
Selection of ssDNA aptamers targeting DNA-bound cJun. **A)** cJun was incubated with the immobilized DNA and the complexes were washed prior to the addition of the ssDNA pool. Six rounds of SELEX were performed with rounds 5 and 6 including a negative selection as illustrated by the dashed arrows. **B)** Four individual sequences from round 6 of the selection, the conserved motifs are highlighted. **C)** EMSAs with aptamer-1, 16, 19, and 27. For each aptamer, either cJun/cJun or cJun/cFos was titrated from 0.4 nM to 6.5 nM.

Our goal was to use DNA SELEX to obtain aptamers that selectively bind cJun/cJun homodimers. Since, AP-1 proteins share a highly conserved bZip domain, responsible for protein dimerization and recognition of the consensus AP-1 element [16], we hypothesized that blocking the DNA-binding domain during the selection process would facilitate obtaining DNA aptamers that have a higher binding affinity for cJun homodimers than cJun heterodimers such as cJun/cFos. cJun was incubated with dsDNA containing a consensus AP-1 site that was immobilized to magnetic beads via a biotin-streptavidin linkage. We selected DNA aptamers from a starting pool of ∼1×10^15^ unique sequences that were 78 nt long, consisting of a 40 nt randomized region flanked with constant regions of two different sequences. A schematic of the SELEX method is detailed in Figure 1A. We performed 6 rounds of positive selection with rounds 5 and 6 also including a negative selection against beads and immobilized DNA. As shown in Figure S1A in File S1, the DNA pool obtained after round 6 showed apparently stronger association with cJun/cJun that with cJun/cFos, despite the fact that cJun/cFos has a higher affinity than cJun/cJun for a consensus AP-1 DNA element (Figure S1B in File S1).

Individual DNA molecules were isolated after round 6 and 46 clones were sequenced. All 46 sequences were different but contained families of over-represented motifs in the 5′ and 3′ ends of the randomized regions; no single sequence motif was present in all the aptamers. We experimentally tested 12 of the 46 sequenced aptamers that were representative of the sequence diversity present across all 46. All 12 aptamers bound cJun/cJun with high affinity. Four aptamers (1, 16, 19, and 27) that were amongst the highest affinity binders were chosen for further experimentation to address specificity for binding cJun/cJun homodimers compared to cJun/cFos heterodimers. Sequence alignment of these aptamers is shown in Figure 1B, aptamers 1, 16, and 19 contain conserved motifs near the 5′ and 3′ ends of the randomized region, while aptamer 27 lacks the 3′ conserved motif. As shown by the EMSA in Figure 1C, all four aptamers bound cJun homodimers with low or sub-nanomolar binding affinity and did not appreciably bind cJun/cFos heterodimers over the concentration range tested. Moreover, the affinity of cJun/cJun homodimers for binding each of these aptamers is greater than the affinity with which cJun/cJun binds a consensus AP-1 element (see Figure S1B in File S1). Because our primary goal was to obtain a single aptamer that bound cJun/cJun with high affinity and selectivity over cJun/cFos, we chose aptamer-19 for further characterization.

### Aptamer-19 has a defined secondary structure, with three distinct elements required for binding cJun/cJun

**Figure 2:**
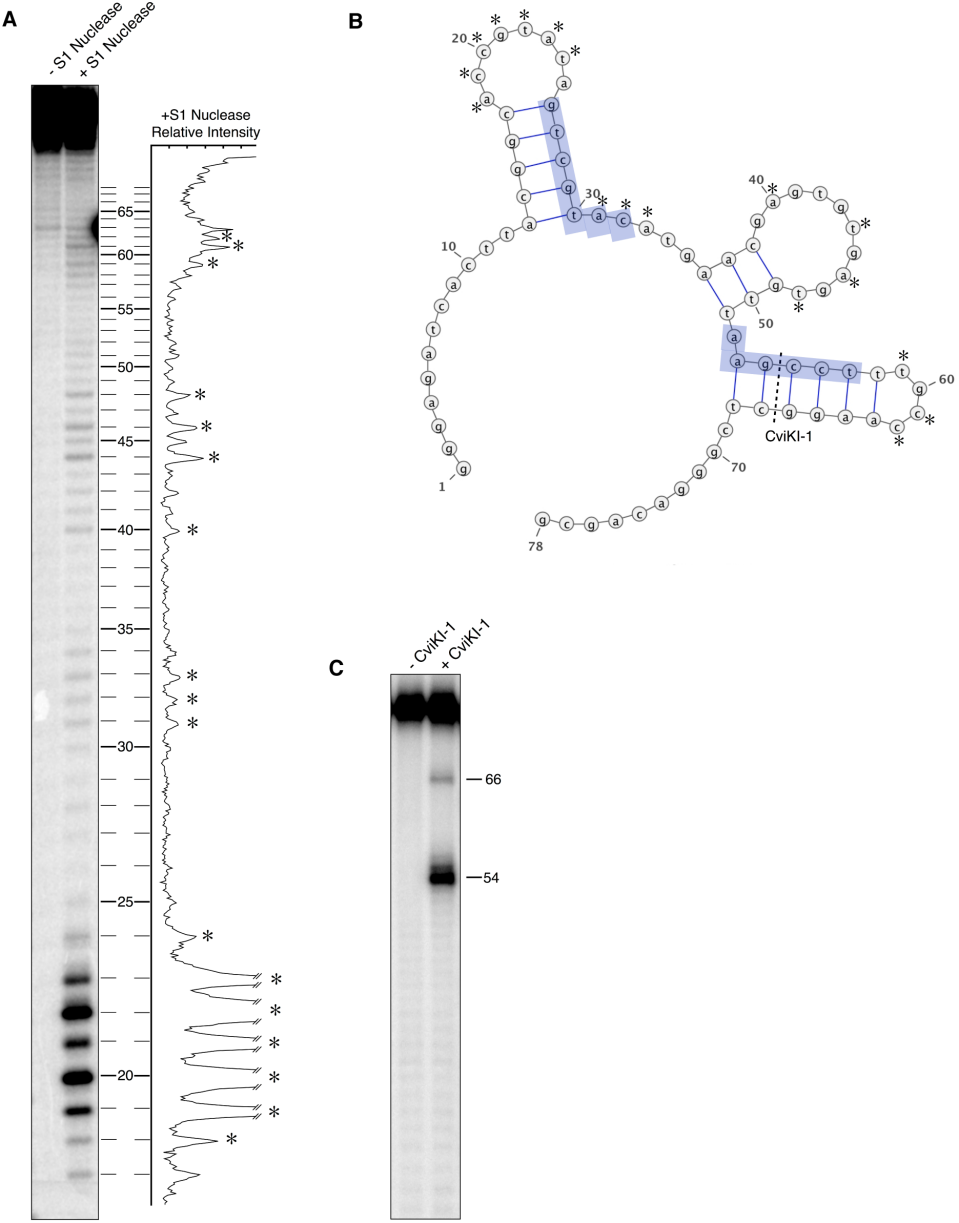
Secondary structure probing of aptamer-19. **A)** ^32^P-labeled aptamer was digested with S1 nuclease and resolved by denaturing gel electrophoresis. The relative band intensities of the +S1 nuclease lane are plotted according to nucleotide position at the right of the gel. **B)** The secondary structure of aptamer-19 as predicted by mFold given the single stranded constraints determined by S1 nuclease digestion. Conserved motifs are highlighted in blue. **C)** Restriction digest with CviKI-1 confirms the presence of the third stem-loop containing the double stranded recognition sequence 5′-AGCC-3′. The CviKI-1 site is labeled in panel B.

To ultimately gain insight into the mechanism of aptamer binding to cJun/cJun, we interrogated the secondary structure of aptamer-19. First, to identify single stranded regions of the aptamer we used S1 nuclease, which preferentially degrades single stranded nucleotides. 5′-^32^P-labeled aptamer-19 was digested with S1 nuclease and the products were resolved by denaturing electrophoresis (Figure 2A). The relative band intensities were determined and plotted with the corresponding nucleotide position. Nucleotides that are predicted to be single stranded by S1 nuclease digestion are indicated by asterisks. To obtain a model for the secondary structure of the apatmer, the single stranded positions were used as input constraints for the mFold secondary structure prediction program (Figure 2B) [17]. The predicted secondary structure contains three stem loops spanning from nucleotides 13–68. Portions of each constant region are predicted to anneal with the conserved motifs in the 5′ and 3′ ends of the randomized region (highlighted in blue) to form the flanking stems.

To further validate the proposed secondary structure we performed a restriction digest of 5′-^32^P-labeled aptamer-19 with CviKI-1, which recognizes the double stranded motif 5′-RGCY-3′. If the third stem is present then CviKI-1 would create blunt-end cuts after nucleotides 54 and 66. As shown in Figure 2C the predominant product created upon digestion with CviKI-1 is a 54 nt fragment, indicating the presence of the third stem-loop from nucleotides 53–68. The less abundant 66 nt product corresponds to CviKI-1 making a single cut between nucleotides 66 and 67.

**Figure 3:**
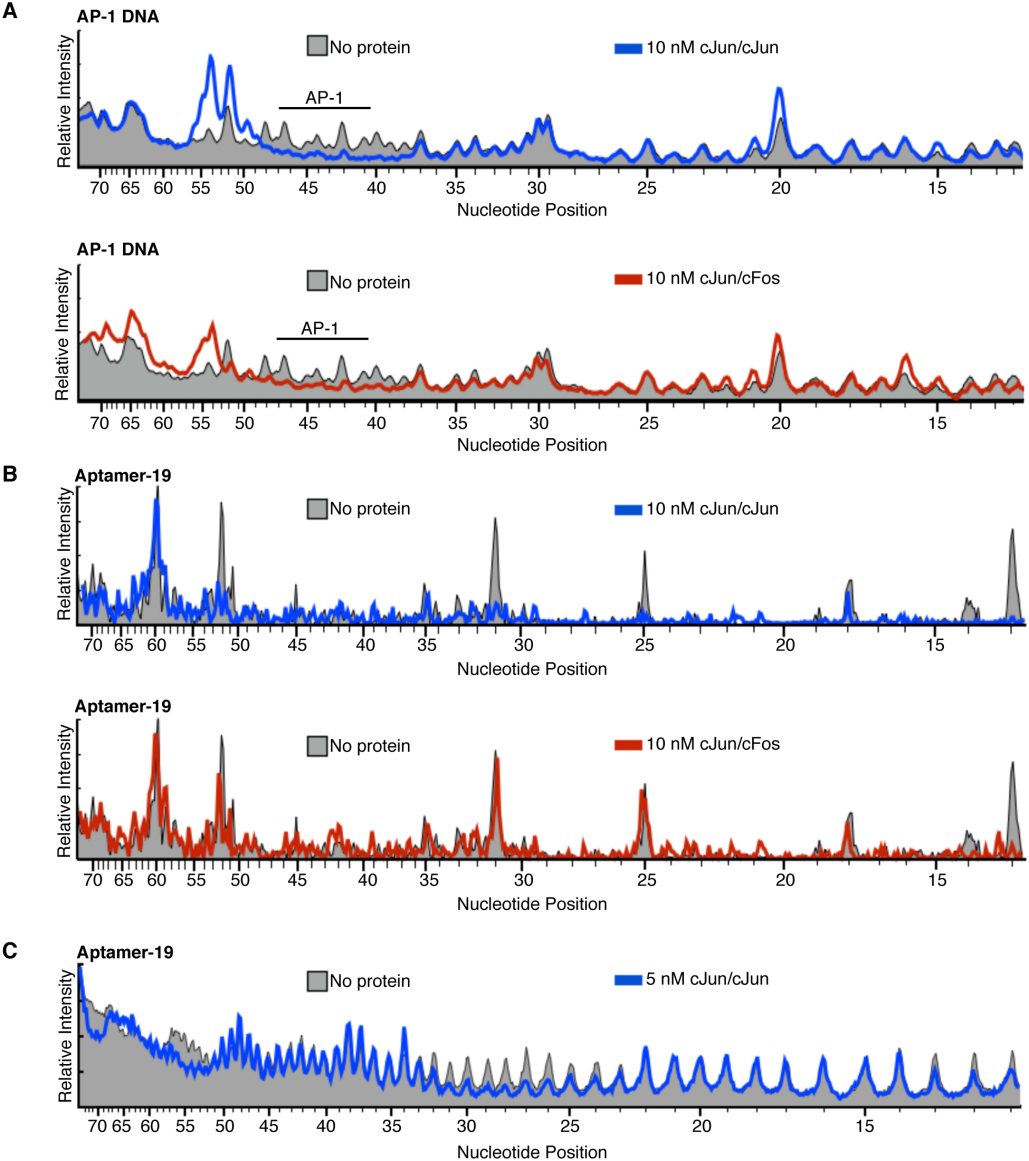
cJun/cJun homodimers, but not cJun/cFos heterodimers make extensive contacts with aptamer-19. **A)** Both cJun/cJun and cJun/cFos protect a discrete region surrounding the AP-1 site on a 78nt long dsDNA construct from DNase I digestion. The relative intensities of the digested products are plotted corresponding to nucleotide position. The digestion profile is presented in the absence of protein (solid gray), in the presence of 10 nM cJun/cJun (blue line), or the presence of 10 nM cJun/cFos (red line). **B)** cJun/cJun broadly protects aptamer-19 from DNase I digestion, while cJun/cFos does not. **C)** Digestion profile of aptamer-19 upon hydroxyl radical cleavage in the absence (solid gray) or presence of 5 nM cJun/cJun (blue line).

To begin to identify the regions of aptamer-19 that interact with cJun we performed DNase I footprinting. As a positive control for DNase I footprinting we designed a dsDNA construct 78 nt long, the same length as the aptamer, with a high affinity AP-1 site from nucleotides 41–47. As shown in Figure 3A both cJun/cJun and cJun/cFos protect a 15 nt region centered around the canonical AP-1 site, with each dimer inducing a unique profile of hypersensitive sites (raw data shown in Figure S2 in File S1). As shown in Figure 3B, DNase I digestion of aptamer-19 without cJun/cJun or cJun/cFos present (sharp grey peaks, raw data shown in Figure S2 in File S1) results in fewer positions that show significant cleavage compared to digestion of the double stranded DNA shown in panel A. Despite less overall cleavage of aptamer-19, cJun/cJun protects nearly all of the cleaved nucleotide positions, revealing a much broader protection profile for aptamer-19 compared to AP-1 DNA. cJun/cFos only protected aptamer-19 near the 5′ end, which further indicates that the aptamer binds cJun/cJun with specificity.

To obtain a higher resolution view of the regions of aptamer-19 that interact with cJun we performed hydroxyl radical footprinting. Hydroxyl radical cleavage targets the backbone of DNA with reactivity that is proportional to the solvent accessibility; as a result, the cleavage products are independent of the primary nucleotide sequence [18]. Aptamer-19 was subjected to hydroxyl radical cleavage in the absence of cJun, and in the presence of 5 nM or 20 nM cJun (Figure S3 in File S1). The digested products were resolved by denaturing electrophoresis and the relative intensities of cleaved aptamer bands were plotted according to the corresponding nucleotide position (Figure 3C). The addition of cJun/cJun protects the aptamer from hydroxyl radical cleavage at 4 distinct regions; nucleotides 11–13, 24–32, 52–56, and 68–72 all show a decrease in cleavage upon addition of cJun. When mapped onto the secondary structure of aptamer-19 these nucleotides occupy portions of the first and third stem-loops as well as the flanking single stranded regions (Figure S4 in File S1, circles). These data are consistent with the DNase I footprinting results (Figure S4 in File S1, triangles). Notably, the conserved motifs within the random region of aptamer-19 are contained within the regions that interact with cJun/cJun. Our results also show that cJun/cJun protects a broader region of aptamer-19 than the AP-1 consensus DNA elements, which suggests that the higher affinity of cJun/cJun for binding the aptamer compared to the consensus AP-1 element is due to a greater number of protein-nucleic acid contacts.

### Aptamer-1\12–74) has high specificity for binding cJun/cJun versus cJun/cFos and blocks cJun/cJun from binding an AP-1 DNA element

**Figure 4:**
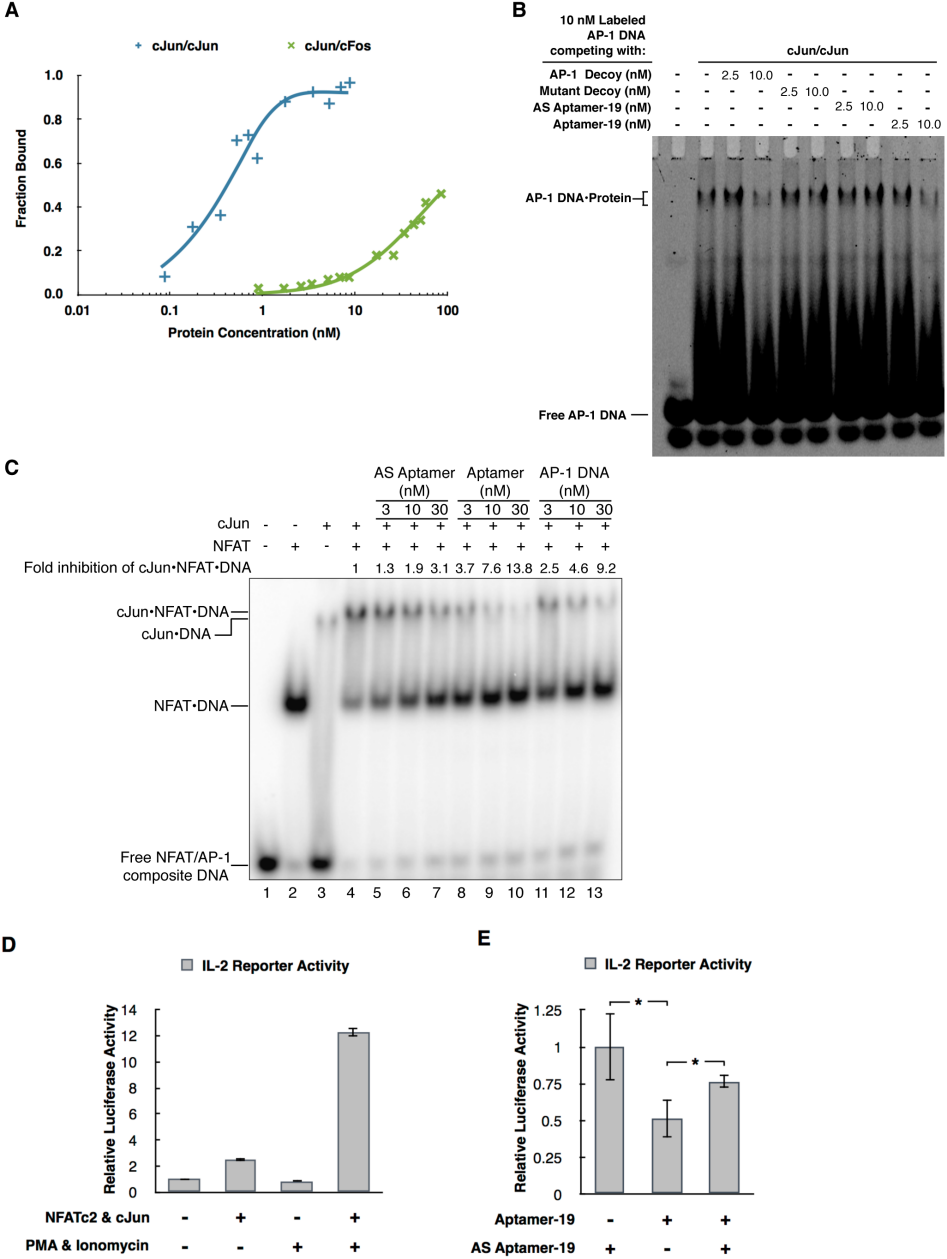
Aptamer-19(12–74) binds cJun/cJun with high affinity and specificity, competes with AP-1 DNA for binding cJun, and inhibits cJun/cJun activated transcription in cells. **A)** Aptamer-19(12–74) is >100-fold more specific for binding cJun/cJun compared to cJun/cFos. Data from EMSAs were quantified and fit to binding curves; the equilibrium dissociation constant of the aptamer for cJun/cJun is 0.5 nM and for cJun/cFos is >85 nM. **B)** 2 nM cJun/cJun was incubated with 10 nM 5′-Cy5-labeled AP-1 DNA and either unlabeled AP-1 decoy DNA, mutant AP-1 decoy, AS aptamer-19(12–74), or aptamer-19(12–74) at the concentrations indicated. **C)** Aptamer-19 prevents cJun/cJun from associating with NFAT bound to an NFAT/AP-1 composite site. dsDNA containing an NFAT/AP-1 composite element can be shifted by NFAT and cJun individually (lanes 2 and 3, respectively) or supershifted as a result of NFAT and cJun cooperativity (lane 4). Either an antisense of aptamer-19(12–74), aptamer-19(12–74), or AP-1 DNA was titrated into the reaction containing NFAT/AP-1 composite DNA, NFAT, and cJun. **D)** Overexpression of NFATc2 and cJun as well as stimulation with PMA and ionomycin are required for full activation of the IL-2 reporter in cells. Firefly luciferase was normalized to renilla luciferase; bars represent the average of three transfections and error bars represent one standard deviation. **E)** Aptamer-19(12–74) reduces transcription driven by cJun/cJun and NFATc2 from the IL-2 reporter in cells. Oligonucleotide concentrations were held constant under all conditions by cotransfecting a scrambled oligonucleotide of the same length. Firefly luciferase was normalized by the firefly luciferase plasmid copy number in each pool of transfected cells, as determined by real time PCR. Data were normalized to the IL-2 reporter activity in the presence of AS aptamer-19(12–74). Bars represent the average of three transfections and error bars represent one standard deviation. Asterisks represent statistical significance determined by a paired t-test. (*, p-value<0.032)

To identify the minimal region of aptamer-19 required to bind cJun/cJun with high affinity we used a series of truncations that systematically removed secondary structural elements (Figure S5 in File S1). These experiments showed the minimal binding region of aptamer 19 to be nucleotides 12–74. Aptamer-19 (12–74) was then used in EMSAs to measure the fold specificity for binding cJun/cJun homodimers over cJun/cFos heterodimers (Figure 4A). Aptamer-19(12–74) bound cJun/cJun homodimers with a K_D_ of 0.5 nM and cJun/cFos heterodimers with a K_D_>85 nM. Hence, there is greater than 100-fold specificity of the aptamer for binding cJun/cJun homodimers over cJun/cFos heterodimers. These data imply that the aptamer targets a protein interface that is at least partially unique to the cJun homodimer.

We asked whether aptamer-19 would compete with AP-1 DNA for binding cJun homodimers; given how the selection was performed we anticipated that cJun/cJun would still bind an AP-1 site in the presence of aptamer-19. We conducted an EMSA, allowing cJun/cJun to choose between ^32^P-labeled AP-1 DNA or unlabeled aptamer-19. As controls we also tested the following in competition with ^32^P-labeled AP-1 DNA: unlabeled AP-1 DNA decoy (an oligonucleotide that forms a hairpin containing the consensus AP-1 site), a mutant AP-1 DNA decoy (folds into the same structure but has the AP-1 site mutated), or a control oligo that is antisense (AS) to aptamer-19. As shown in Figure 4B, aptamer-19 blocked cJun homodimers from binding AP-1 DNA, which we examine in more detail in the Discussion. The consensus and mutant AP-1 decoys functioned as expected, either blocking or not blocking cJun homodimers from binding DNA, respectively. Importantly, the AS aptamer control did not show any significant inhibition of cJun binding AP-1 DNA.

Transcriptional regulation by AP-1 proteins often involves cooperative binding at composite promoter DNA sites with other transcription factors such as those in the NFAT family of transcriptional activators [19]. At these composite sites, AP-1 and NFAT transcriptional activators cooperate to form a more stable DNA-bound complex. We hypothesized that due to the high binding affinity of aptamer-19 and its broad range of contacts with cJun homodimers, it would more effectively inhibit cJun homodimers from cooperatively binding DNA with NFAT compared to an AP-1 decoy. To test this we performed EMSAs using a high affinity NFAT-AP-1 composite element and asked whether aptamer-19 blocks the formation of the ternary NFAT/cJun/DNA complex. As shown in Figure 4C, NFAT and cJun homodimers individually shifted the composite DNA element (lanes 2 and 3, respectively). Together, NFAT and cJun homodimers cooperatively bound DNA resulting in a slower migrating (supershifted) complex (lane 4). Either the antisense (AS) aptamer (lanes 5–7), aptamer-19 (lanes 8–10), or AP-1 DNA (lanes 11–13) were added at the concentrations indicated. Aptamer-19 showed the sharpest fold-decrease in the amount of NFAT/cJun/DNA complex, hence was more effective at inhibiting cJun homodimers from cooperatively binding DNA with NFAT than was the AP-1 DNA decoy. Additionally, when the cJun/NFAT/DNA complex was lost upon addition of aptamer-19, there was a corresponding increase in the NFAT/DNA band, demonstrating that aptamer-19 specifically targets cJun/cJun and not NFAT. The AS aptamer did not inhibit formation of the ternary complex to nearly the same extent as either the AP-1 decoy or aptamer-19.

### Aptamer-19 inhibits IL-2 reporter activity in cells

We previously reported that cJun homodimers and NFATc2 can achieve high levels of transcriptional synergy on the IL-2 promoter [20]. The high level of synergy requires a unique interaction between the DNA-binding domain of cJun homodimers and the C-terminal activation domain of NFATc2 [5]. We asked if the minimal binding domain of aptamer-19, which can specifically prevent cJun homodimers from binding DNA, could repress cJun/cJun-specific transcriptional synergy with NFATc2 at the IL-2 promoter. Figure 4D illustrates transcriptional activation of an IL-2 reporter in response to overexpression of NFATc2 and cJun, and stimulation by PMA and ionomycin. Maximal IL-2 activity is only achieved when NFATc2 and cJun are overexpressed and cells are stimulated with PMA and ionomycin. When cotransfected under these conditions, aptamer-19 repressed IL-2 luciferase activity relative to its antisense sequence (Figure 4E). Moreover, the repression mediated by aptamer-19 was attenuated by cotransfecting an equal amount of its antisense sequence, acting in essence as an antidote for aptamer-19. Thus, aptamer-19 appears to inhibit cJun homodimer-mediated transcription in cells. These results establish the potential for AP-1 dimer specific aptamers to serve as useful tools in dissecting the role of AP-1 dimer composition in transcriptional regulation.

## Discussion

AP-1 transcription factors are key regulators of proliferation, tumorigenesis, apoptosis, and the immune response. Individual AP-1 family members are differentially expressed, resulting in a complex mixture of AP-1 proteins that are likely context and cell-type specific. Here, we used SELEX to identify DNA aptamers that bind cJun/cJun with high affinity and specificity compared to cJun/cFos. Biochemical assays revealed the secondary structure of an aptamer, the minimal region necessary for binding cJun homodimers, and showed that the aptamer competes with AP-1 DNA for binding cJun/cJun. In cells, the aptamer repressed cJun homodimer activated transcription from a reporter. This work sets the foundation and provides a selection method for interrogating the biological functions unique to distinct AP-1 dimer compositions.

### An aptamer that binds cJun/cJun with high affinity and specificity

The SELEX procedure has been used to isolate RNA and DNA aptamers that bind an extensive catalog of protein targets with high affinity and specificity [21]. In particular, aptamers have proved to be extremely useful tools in dissecting the role of specific protein-protein and protein-DNA interactions of various transcription factors [22]–[26]. In many of these cases the selected aptamers bind where DNA typically interacts, which is a high affinity nucleic acid binding site. It has been demonstrated, however, in a selection against the TATA-binding protein (TBP), that by masking the DNA binding domain aptamers can be directed to discrete binding surfaces on the protein [25]. Here, we chose to mask the DNA binding domain of cJun homodimers with AP-1 DNA during the selection, with the goal of directing the aptamers to bind a surface on cJun homodimers that is not present on cJun/cFos heterodimers.

Given how we designed our selection process, however, we were surprised to find that aptamer-19 blocked cJun from binding AP-1 DNA (Figure 4B). A likely model for this is that the region(s) of the aptamer not responsible for specific cJun recognition could, driven primarily by electrostatic interactions, occupy the basic region of the cJun DNA binding domain, thereby preventing it from subsequently recognizing AP-1 DNA. During the selection, when cJun/cJun was pre-bound to AP-1 DNA, specific high affinity contacts with cJun outside the DNA binding domain were adequate to recover aptamers. This model is consistent with the observation that during the SELEX the last aptamer pool did not displace cJun/cJun from immobilized AP-1 DNA, but aptamer-19 competes with AP-1 DNA when cJun/cJun is given the choice of which DNA to bind. In addition, this binding model suggests that the aptamer binds more than one site on cJun, which is consistent with the broad range of contacts between the aptamer and cJun homodimers as revealed by hydroxyl radical and DNase I footprinting (Figures 3B and 3C). The DNase I footprint of DNA containing an AP-1 site showed a significantly smaller region of protection by cJun/cJun compared to the aptamer DNA (Figure 3A). Moreover, the ∼100-fold specificity for binding cJun homodimers compared to cJun/cFos heterodimers supports a binding mechanism in which the aptamer associates with regions of cJun/cJun outside the DNA binding domain.

cJun/cJun binds aptamer-19 with a higher binding affinity compared to the consensus AP-1 sequence (∼0.5 nM versus ∼15 nM, respectively). Aptamers that target the DNA binding domain of transcription factors tend to have a binding affinity similar to, or weaker than, the optimal DNA recognition site. For example, heat shock factor 1 (HSF-1) binds its respective DNA recognition element with a K_D_ of ∼1 nM, yet binds an RNA aptamer isolated after 14 rounds of SELEX with an affinity of 20–40 nM [23], [27]. Similarly, RNA aptamers targeting the DNA binding domain of NFATc2 bind with a K_D_ of ∼10–100 nM, which is approximately 10-fold weaker than the NFAT DNA recognition sequence [28], [29]. One of the most widely studied transcription factor aptamers targets NFkB and inhibits NFkB from binding DNA [30]. This RNA aptamer has a similar binding affinity for NFkB as the DNA recognition element, and crystal and NMR structures revealed that this aptamer structurally mimics the NFkB DNA recognition element [31], [32]. By contrast, the observations that aptamer-19 binds cJun/cJun with at least 30 fold greater affinity than a consensus AP-1 DNA site and that the aptamer can block DNA binding by cJun/cJun is consistent with the aptamer interacting with cJun/cJun in the DNA binding domain as well as another region of the protein.

It was interesting to find that both of the constant regions used during the selection formed stem-loop structures with sequences derived from the randomized region in aptamer-19. We found these two stem-loops were required for binding cJun/cJun (Figure S5 in File S1), although in both cases we do not know whether it is the structure or the sequence of the stem-loops that is important for mediating binding. This raises the question of whether using a different set of constant regions would also result in selecting high-affinity aptamers with critical stem-loops forming between sequences in the constant and randomized regions. If the primary determinant of high affinity binding is indeed the structures and not the sequences of the flanking stem-loops (see Figure 2B), then choosing a different constant region would likely result in selection of aptamers that still contain sequences complementary to the constant region in order to form two flanking stem-loops. Alternatively, if both sequence and structure of the flanking stem-loops in aptamer-19 are important for binding, then performing the selection with a different constant region has the potential to result in aptamers that bind cJun/cJun with a different structure from that of aptamer-19.

### The potential for aptamers to delineate AP-1 activation mechanisms in cells

At the promoters of inducible genes, such as those involved in the immune response, AP-1 proteins cooperate with other transcription factors, namely those in the NFAT family. Given that aptamer-19 has higher affinity for cJun homodimers than AP-1 DNA we hypothesized that this aptamer could be an effective inhibitor of cJun/NFAT cooperative DNA binding. By EMSA, we showed that aptamer-19, but not its antisense sequence, significantly inhibited cJun/cJun from cooperatively binding DNA with NFAT.

Since aptamer-19 functioned to potently inhibit cJun from binding AP-1 DNA, we asked if it could inhibit transcription from an IL-2 promoter in cells. We previously reported that cJun homodimers and NFATc2 cooperate to drive high levels of synergistic transcription from the IL-2 promoter [5], [20]. Since these high levels of synergy are unique to cJun homodimers, this allowed us to test for repression of cJun/cJun activated transcription. By transient transfection, we found that aptamer-19 repressed cJun/cJun and NFAT-dependent IL-2 reporter activity compared to transfection with an AS control oligo. Moreover, the repression by aptamer-19 was attenuated by cotransfection with the antisense control oligo, suggesting that the repression mediated by aptamer-19 necessitates the defined secondary structure of the aptamer.

Recent studies have identified a potential coactivator function unique to cJun homodimers but not cJun/cFos heterodimers [3]–[5]. In this context, cJun utilizes its DNA binding domain to interact with DNA bound transcription factors. By specifically targeting cJun/cJun and obstructing DNA binding, aptamer-19 could prove to be a useful tool in probing the biological functions of cJun homodimers, including its role as a coactivator. We believe that this experimental approach also sets the foundation for targeting various AP-1 dimer compositions, helping to discern their distinct biological roles.

## Supporting Information

File S1 **Figure S1,** Relative aptamer and AP-1 DNA binding affinities of cJun/cJun homodimers and cJun/cFos heterodimers. **Figure S2,** DNase I footprinting of AP-1 DNA and aptamer-19. **Figure S3,** Hydroxyl radical cleavage of aptamer-19 shows four distinct regions of protection upon addition of cJun. **Figure S4,** cJun footprint determined by DNase I and hydroxyl radical cleavage mapped onto the secondary structure of aptamer-19. **Figure S5,** All three stem-loops of aptamer-19 are required for binding cJun/cJun with high affinity.
(PDF)
