# Supplementary material for: Selection and Characterization of a DNA Aptamer That Can Discriminate between cJun/cJun and cJun/cFos
Source: PLoS One. 2014 Jun 26;9(6):e101015. doi: 10.1371/journal.pone.0101015 (PMC4072727; doi:10.1371/journal.pone.0101015)
Supplement: File S1 — Figure S1, Relative aptamer and AP-1 DNA binding affinities of cJun/cJun homodimers and cJun/cFos heterodimers. Figure S2, DNase I footprinting of AP-1 DNA and aptamer-19. Figure S3, Hydroxyl radical cleavage of aptamer-19 shows four distinct regions of protection upon addition of cJun. Figure S4, cJun footprint determined by DNase I and hydroxyl radical cleavage mapped onto the secondary structure of aptamer-19. Figure S5, All three stem-loops of aptamer-19 are required for binding cJun/cJun with high affinity. (PDF) [file pone.0101015.s001.pdf]

Figure S1

A

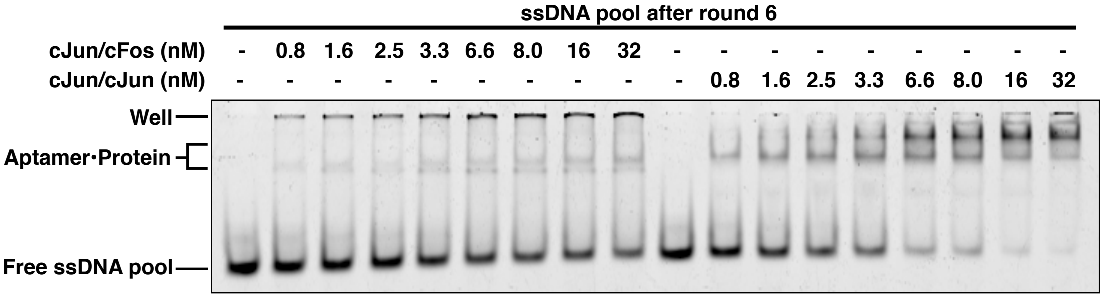

B

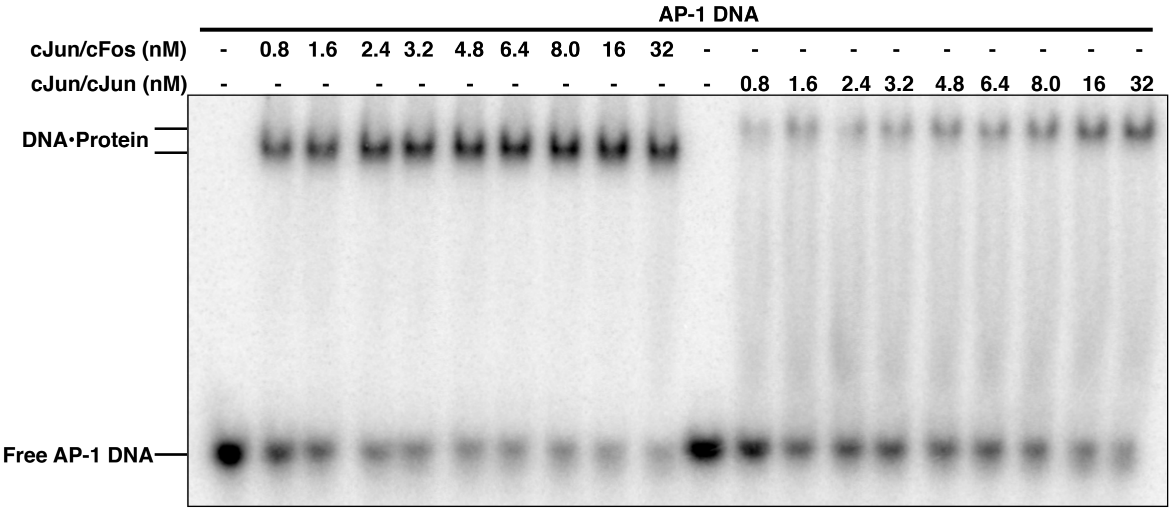

Figure S1. Relative aptamer and AP-1 DNA binding affinities of cJun/cJun homodimers and cJun/cFos heterodimers. A) An EMSA shows that the DNA isolated after the final round of SELEX binds cJun homodimers with significantly higher affinity than cJun/cFos heterodimers. **B)** An EMSA with ^{32}P-labeled AP-1 DNA containing the canonical TGA(C/G)TCA recognition element and a titration of both cJun/cFos heterodimers and cJun/cJun homodimers shows that cJun/cFos has a higher binding affinity than cJun/cJun.

Figure S2

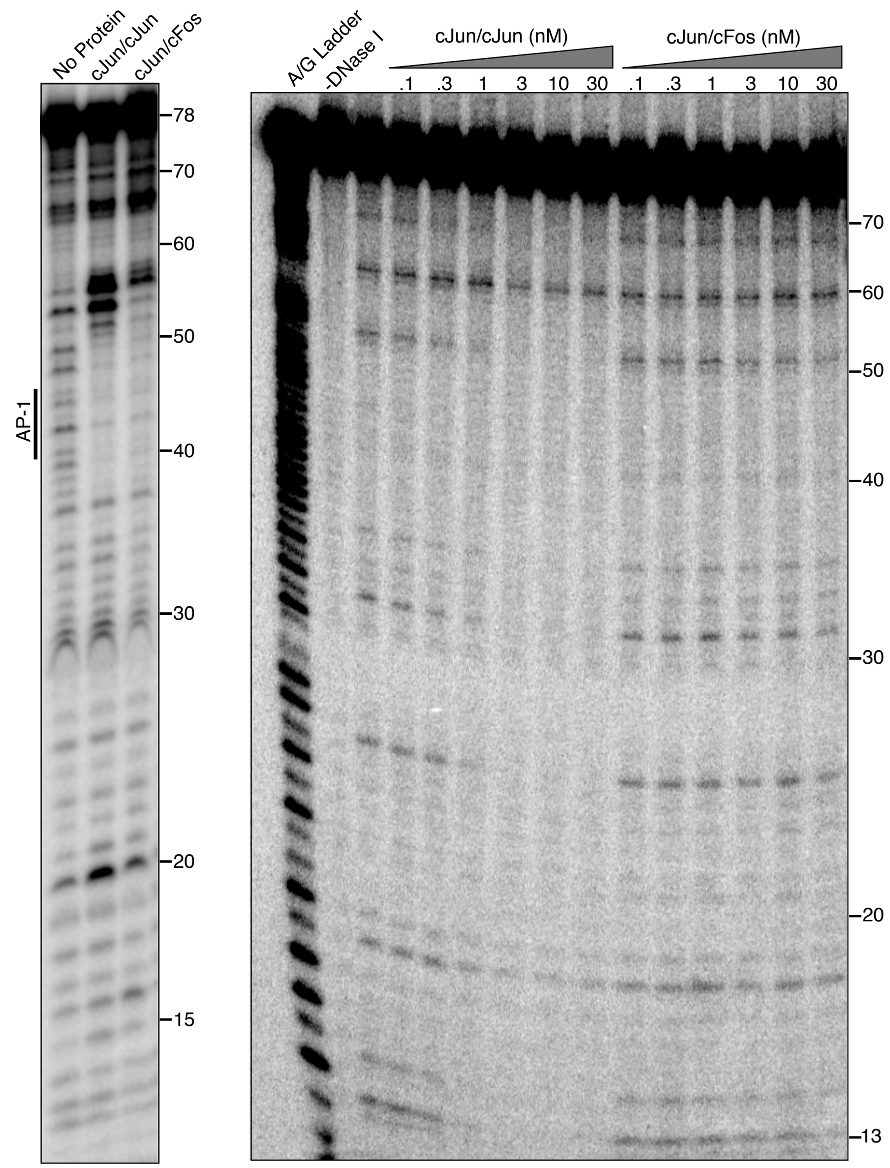

Figure S2. DNase I footprinting of AP-1 DNA and aptamer-19. A) Footprinting 78 nt long dsDNA containing a consensus AP-1 site at nucleotides 41-47 in the presence of either cJun/cJun or cJun/cFos. Both dimers protect ~15 nt surrounding the AP-1 element. **B)** Footprinting of aptamer-19 reveals a broad range of protection with the addition of cJun/cJun but not cJun/cFos.

Figure S3

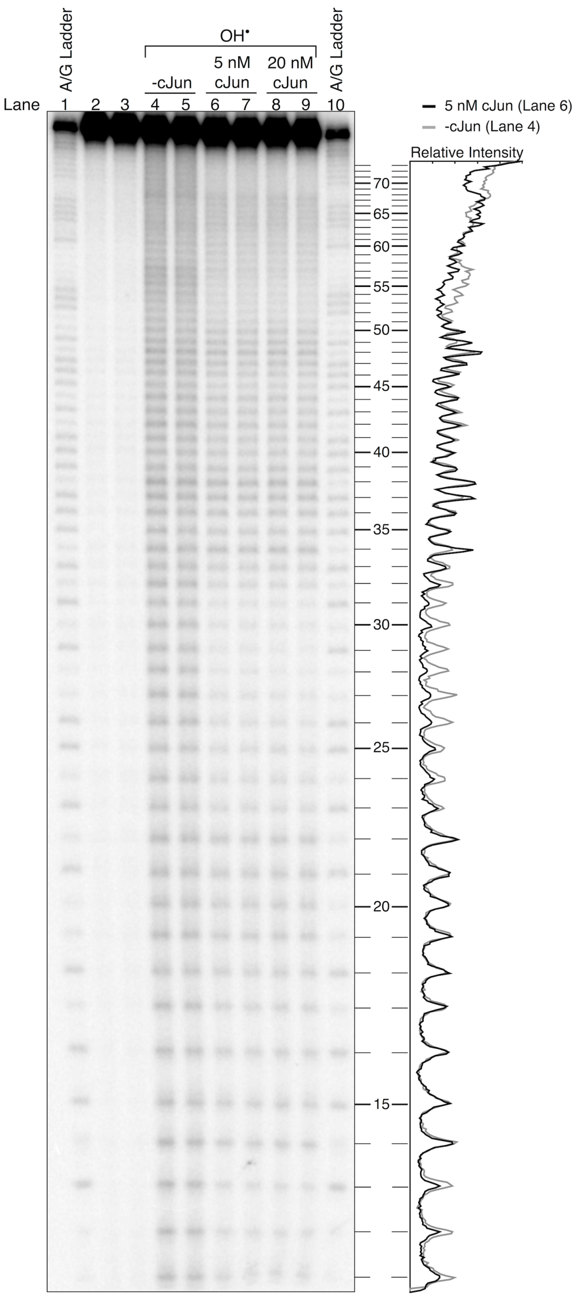

Figure S3. Hydroxyl radical cleavage of aptamer-19 shows four distinct regions of protection upon addition of cJun. Aptamer-19 was subjected to hydroxyl radical cleavage in the absence of cJun (lanes 4, 5), with 5 nM cJun (lanes 6, 7), or with 20 nM cJun (lanes 8, 9). The nucleotide position and the relative intensities of the cleavage products in the absence of cJun (lane 4, gray trace) and in the presence of 5 nM cJun (lane 6, black trace) are positioned to the right of the gel image.

Figure S4

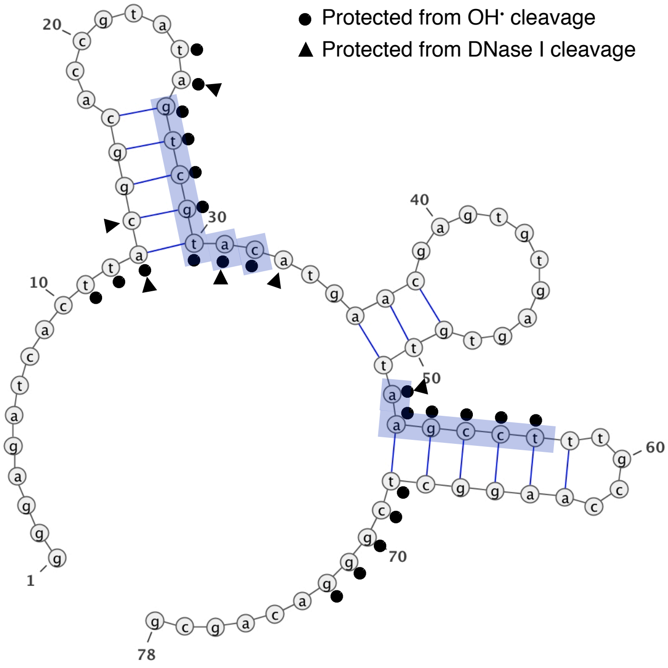

Figure S4. cJun footprint determined by DNase I and hydroxyl radical cleavage mapped onto the secondary structure of aptamer-19. Circles indicate the footprint identified by hydroxyl radical cleavage and triangles represent the nucleotides protected by DNase I cleavage. Conserved motifs are highlight in blue.

Figure S5

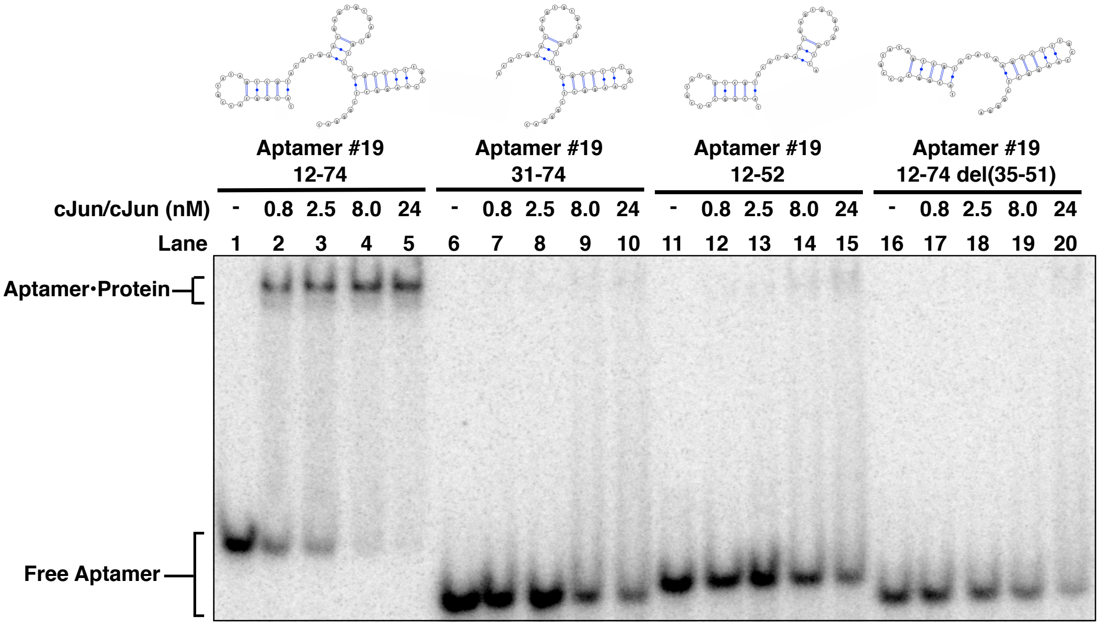

Figure S5. All three stem-loops of aptamer-19 are required for binding cJun/cJun with high affinity. Truncations were made eliminating portions of the constant regions (12-74), deletion of the first stem-loop (31-74), third stem-loop (12-52), or the middle stem-loop (12-74 with a deletion from 35-51). Each truncation was tested for binding cJun by EMSA.
